# Urban Populations

**Published:** 1903-07-18

**Authors:** 


### Urban Populations.

The recent debate in the House of Lords, on the alleged physical degeneracy of urban populations, deals with a question which the principal speakers rightly described as of imperial rather than merely of national importance. The Earl of Meath, with whom the discussion originated, based his observations chiefly upon the report of the Royal Commission on Physical Training in Scotland, and on that of the Inspector-General of Recruiting for 1902, in which it was said that the one subject which causes anxiety in the future as regards recruiting is the gradual deterioration of the physique of the working classes from which the bulk of the recruits must always be drawn. The Bishop of Ripon, who followed on the same side, endeavoured to show that the classes in question were also failing to maintain their proper rate of increase, and expressed his fear that the diminution of numbers to which he called attention pointed, not to self denial, but to self-indulgence and the restriction of population by artificial means. The Duke of Devonshire, on the part of the Government, said that the matter was engaging the attention of his colleagues, and that steps had already been taken to obtain the assistance of the Royal Colleges of Physicians and Surgeons as advisers with regard to some of its aspects. He quoted certain statistics of a more favourable character than those referred to by previous speakers, expressed his opinion that the questions of possible degeneration and of diminished increase must be kept apart for any purposes of investigation, and announced the intention of the Government to appoint a commission of inquiry as soon as the scope of its labours could be defined.

We are strongly of opinion that the physique of the townsman is better now than at any former period of urban history, and that it is in course of improvement rather than in course of deterioration. There are many reasons why this should be the case, reasons furnished by better housing, better water supply, better drainage, better wages and food, and, above all, by the great body of protective legislation summed up under the general designation of the Factory Acts. Only 63 years have passed away since the publication of Mrs. Trollope's novel, "Michael Armstrong, or the Factory Boy." It was illustrated, and the illustrations were not thought extravagant at the time of their appearance. Anyone who has not seen them might employ time profitably in hunting up the book on the shelves of any library of sufficient antiquity, and he would be rewarded by the attainment of quite new views on the subject of the physique of urban populations.

We cannot, therefore, unreservedly accept the authorities quoted by the Earl of Meath and the Bishop of Ripon; but, at the same time, we have no desire to minimise the importance of the question at issue, which is well worthy of all the attention which Commissions or the Legislature can bestow upon it. We fully agree with Lord Rosebery that the maintenance of an imperial race is necessary to the preservation of empire, and we should welcome the employment of every means by which the causes of possible physical deterioration could be pointed out for avoidance. Some of them are probably not far to seek. A prominent place among these may perhaps be given to the almost universal ignorance of cookery among women of the working classes, an ignorance which leads not only to the waste of much good and wholesome food, but also to the purchase and consumption of a variety of non-nutritious or unwholesome messes, many of them recommended to the credulity of the ignorant by mendacious advertisements. The bicycle has probably been an influence for good; but the so-called love of sport which induces thousands of men and boys to go and look on while others play cricket or football has no tendency towards the promotion of physical welfare. The elaborate investigations made at some of the American universities seem to leave no doubt that tobacco is highly prejudicial to boys and adolescents, and a city boy of the lower orders without a cigarette is now almost a rarity, insomuch that it may be fairly questioned whether smoking is not a greater national evil than alcoholic intemperance. It is unquestionably practised more universally and also earlier in life, at a time, therefore, when its worst effects are most certain to be realised. The school ought to be, and we trust soon will be, the means by which some at least of the influences most prejudicial to the mental and physical welfare of the rising generation may be brought under control; and, in the meanwhile, it will be the part alike of philanthropy and of patriotism to promote the growth and popularity of the clubs, societies, and other kindred institutions, by which so many of the youth of the lower classes are now being guided into the paths of manliness and of good conduct.

